# Odor Identification and Regional Gray Matter Atrophy in Patients with Alzheimer’s Disease, Parkinson’s Disease, and the Healthy Elderly: A Cross-Sectional Structural MRI Study

**DOI:** 10.3390/brainsci11101296

**Published:** 2021-09-29

**Authors:** Simonas Jesmanas, Rymantė Gleiznienė, Mindaugas Baranauskas, Vaidas Matijošaitis, Daiva Rastenytė

**Affiliations:** 1 Department of Radiology, Medical Academy, Lithuanian University of Health Sciences, Mickevičiaus Str. 9, LT-44307 Kaunas, Lithuania; simonas.jesmanas@stud.lsmu.lt; 2 Biomedical Engineering Institute, Kaunas University of Technology, K. Baršausko Str. 59-454, LT-44029 Kaunas, Lithuania; m.baranauskas@ktu.lt; 3 Department of Neurology, Medical Academy, Lithuanian University of Health Sciences, Mickevičiaus Str. 9, LT-44307 Kaunas, Lithuania; vaidas.matijosaitis@lsmuni.lt; (V.M.)daiva.rastenyte@lsmuni.lt (D.R.)

**Keywords:** odor identification, Alzheimer’s disease, Parkinson’s disease, gray matter atrophy, orbitofrontal cortex

## Abstract

Multiple associations between impaired olfactory performance and regional cortical and deep gray matter atrophy have been reported in separate studies of patients with Alzheimer’s disease (AD), Parkinson’s disease (PD), and of the healthy elderly. We aimed to evaluate such possible associations among these populations in a unified manner. Twenty AD, twenty PD patients’ and twenty healthy age- and sex-matched controls’ odor identification performance was assessed with the Lithuanian adaptation of the Sniffin’ Sticks 12 odor identification test, followed by morphometric gray matter analysis by MRI using FreeSurfer. AD patients had significantly lower cognitive performance than both PD patients and the healthy elderly, as evaluated with the Mini-Mental State Examination (MMSE). Odor identification performance was significantly worse in AD and PD patients compared with the healthy elderly; AD patients performed slightly worse than PD patients, but the difference was not statistically significant. Among patients with AD, worse odor identification performance was initially correlated with atrophy of multiple cortical and deep gray matter regions known to be involved in olfactory processing, however, only two measures—decreased thicknesses of the right medial and left lateral orbitofrontal cortices—remained significant after adjustment for possible confounders (age, MMSE score, and global cortical thickness). Among patients with PD and the healthy elderly we found no similar statistically significant correlations. Our findings support the key role of the orbitofrontal cortex in odor identification among patients with AD, and suggest that correlations between impaired odor identification performance and regional gray matter atrophy may be relatively more pronounced in AD rather than in PD.

## 1. Introduction

Olfactory function can be divided into odor detection, discrimination, and identification [1,2]. Odor detection is the most basic form of olfaction which entails detection of an olfactory stimulus at a particular threshold; worse odor detection performance indicates a higher odorant concentration needed for its perception [1]. Odor discrimination is a higher-order task which entails comparing odors and perceiving their difference or similarity in quality or strength [1]. Odor identification is the most complex olfactory task, which entails correctly identifying a specific odor either by selecting it from a list of possible odors or naming it without options [1]. Olfactory performance decreases with age; one study of people older than 53 showed that olfactory impairment was present in almost 25% [3]. Age-related olfactory decline may be associated with levels of exercise and overall physical activity [4]. Also, olfactory performance and its age-related decline, at least with respect to odor detection (odor perception threshold), may not be uniform across different age groups, as it was shown to have several distinct phenotypes (juvenile, adult, and elder) [5]. Furthermore, cognitive impairment and neurodegenerative diseases negatively influence olfactory function: Alzheimer’s disease (AD) significantly impairs higher-order olfactory processing (odor discrimination and identification) with a slightly weaker effect on lower-order olfactory processes (odor detection), while Parkinson’s disease (PD) impairs both higher-order and lower-order olfactory processes more homogenously; thus, deficits in odor detection are relatively more evident in patients with PD [1,6,7]. This slightly different nature of olfactory dysfunction can be in part explained by the greater cognitive and semantic deficits noted in most cases of AD, which may contribute to preferential impairment of higher-order olfactory processing rather than the cognitively simpler odor detection [1,6]. Despite these slight differences all facets of olfactory dysfunction were recognized as some of the earliest manifestations of neurodegeneration in both AD and PD, and they are useful in evaluating suspected early neurodegeneration, its differential diagnosis, and prognosis [8,9]. Other causes of impaired olfaction include upper respiratory tract infections, sinonasal disease, and head trauma [10].

While the peripheral olfactory system (olfactory epithelium and olfactory bulb) is well described [11], the structural and functional organization of the more central higher-level olfactory pathways is more complex and less well understood [12]. In recent studies the functional connectivity of the human olfactory system was shown to be widespread and include the anterior olfactory nucleus, olfactory tubercle, amygdala, piriform cortex, orbitofrontal cortex, gyrus rectus, insula, anterior cingulate cortex, hippocampus, parahippocampal gyrus, entorhinal cortex, rostral temporal lobe, temporal pole and inferior temporal gyrus, medial prefrontal cortex, Broca’s area, pallidum, caudate nucleus, and putamen; the primary olfactory areas are most often said to include the anterior olfactory nucleus, olfactory tubercle, piriform cortex, and amygdala, while some regions, such as the orbitofrontal and entorhinal cortices, are variably described as either primary or secondary olfactory areas [12,13,14,15,16]. The slight variability of the reported regions in different studies reflects that while some of them are definite key parts of the olfactory pathway (for example, the piriform, orbitofrontal, and entorhinal cortices), others may be variably included due to the widespread nature of higher-order olfactory processing and possibly due to different methods of structural and functional evaluation employed in each attempt to analyze the olfactory system.

Multiple studies of different populations described various relationships between impaired olfactory performance and structural changes in the cortical and deep gray matter. Patients with impaired olfaction due to non-neurodegenerative causes had decreased gray matter volume in the piriform cortex, orbitofrontal cortex, nucleus accumbens, medial and dorsolateral prefrontal cortex, anterior and middle cingulate cortices, insular cortex, hippocampus, parahippocampal gyrus, and cerebellum [17,18]. Among patients with AD, diminished olfactory performance was shown to be associated with atrophy of the primary olfactory cortex, parahippocampal and entorhinal cortices, left precentral and inferior frontal gyri, overall medial temporal lobe, and reduced left hippocampal volume [16,19,20,21,22,23]. Among patients with PD, decreased olfactory performance was reported to be associated with lower gray matter volumes in the piriform and orbitofrontal cortices, amygdala, left putamen, right caudate, and right thalamus [24,25,26,27,28]. Most of these studies point to similar positive relationships between olfactory performance and gray matter morphometric measures. However, one group of researchers reported findings of an opposite nature, namely that in non-demented PD patients olfactory performance was negatively correlated with gray matter and intracerebral volume, and with right caudate and left putamen gray matter density; furthermore, among patients with better olfactory performance there was lower gray matter density in the orbitofrontal, mesiofrontal, prefrontal, temporal, and cingulate areas than among those with worse olfactory performance; to explain these findings researchers invoked the cognitive reserve hypothesis [29]. Cognitive reserve and brain integrity measures are usually negatively correlated in patients with neurodegenerative diseases, but usually positively correlated in the healthy elderly [30]. In this context, the findings that non-demented patients with PD who exhibited better olfactory performance had lower gray matter density in certain regions suggested that preserved olfaction may represent the presence of compensatory neural and cognitive mechanisms that counteract the existing structural atrophy, possibly by better functional connectivity.

While most mentioned studies point to similar brain regions being associated with olfactory dysfunction in these populations, there are multiple discrepancies as the regions involved in these relationships do not completely overlap. Consistently reported associations between olfactory performance and morphometric measures of certain regions support their central role in olfactory processing, while other regions may be variably reported due to their more peripheral place in the various olfactory pathways, differences in applied methods, and differences in particular samples of studied populations. Indeed, separate studies of these populations often used different olfactory tasks and performed structural quantitative analysis with varying tools and protocols.

To our knowledge, specifically, associations between odor identification performance and regional cortical and deep gray matter atrophy among patients with AD, patients with PD, and the healthy elderly were not yet evaluated in a unified manner within a single study. Therefore, in this study we aimed to evaluate the associations between performance of an identical odor identification task and regional cortical and deep gray matter atrophy among these three groups.

Our hypothesis is two-fold: (1) odor identification performance will be significantly worse in the AD group than both PD and control groups, and significantly worse in the PD group than the control group; (2) in the AD and PD groups there will be similar significant positive correlations between odor identification performance and gray matter morphometric measures in regions most strongly associated with olfactory function.

## 2. Materials and Methods

### 2.1. Subjects

Twenty Alzheimer’s disease (AD) and twenty Parkinson’s disease (PD) adult patients were prospectively recruited by neurologists working within the Department of Neurology at the Lithuanian University of Health Sciences Kaunas Clinics from March 2018 until March 2019.

AD patients were included if they: (1) were diagnosed with possible sporadic AD based on the National Institute of Neurological and Communicative Disorders and Stroke, and the Alzheimer’s Disease and Related Disorders Association criteria [31]; (2) were eligible for MRI. PD patients were included if they: (1) were diagnosed with idiopathic or familial PD based on the United Kingdom Parkinson’s Disease Society Brain Bank clinical diagnostic criteria [32]; (2) were eligible for MRI. Twenty age- and sex-matched healthy elderly control subjects were recruited from healthy relatives of included patients.

Subjects were not included if they: (1) refused to participate; (2) had an uncertain diagnosis; (3) had a history of severe head trauma, sinusitis or were currently suffering from an upper respiratory tract infection; (4) had major somatic (severe heart failure, terminal renal or hepatic insufficiency, cancer, hemodynamically significant intracranial/extracranial artery stenosis or occlusion) or psychiatric (psychosis, major depression) disease; (5) were on long-term medications affecting cognitive function; or (6) had significant neurological symptoms (severe visual loss, paresis, aphasia).

### 2.2. Clinical Testing

Subjects were evaluated with the Mini Mental State Examination (MMSE). MMSE provides a measurement of cognitive function, and is scored from 0 to 30, with higher scores representing better cognitive performance [33].

### 2.3. Odor Identification Testing

Subjects were evaluated with the adapted and validated Lithuanian version of the Sniffin’ Sticks 12 (SS12) odor identification test [34]. The test is comprehensively described in the mentioned study [34]. It entails presenting a subject with 12 pen-sticks containing various culturally recognizable odors. The cap of the pen is removed for up to 4 s and positioned about 2 cm in front of the subjects’ nostrils, then the subject is asked to identify the correct odor descriptor from a set of four descriptors written on an answer sheet. The procedure is repeated for all 12 pen-sticks. The test is scored from 0 to 12 based on the number of correct odor identifications.

### 2.4. Magnetic Resonance Imaging

#### 2.4.1. Acquisition

MRI was performed on a 1.5T Siemens MAGNETOM Avanto (Germany) scanner which was not modified or upgraded during the study period. The protocol included standard sequences without contrast enhancement: axial T2W/TSE (TR 4740 ms, TE 3.37 ms, TI 1100 ms, flip angle 120), T2W/fl2d/hemo (TR 800 ms, TE 26 ms, flip angle 20), coronal T2W/TSE (TR 5000 ms, TE 93 ms, flip angle 150), axial DW/ADC (TR 3000 ms, TE 89 ms), axial and coronal T2W/FLAIR (TR 9000 ms, TE 98 ms, TI 2500 ms, flip angle 150), and sagittal T2W/spc2d/iso (TR 3200 ms, TE 379 ms). The sequence used for further volumetric processing was isotropic T1W/mpr/p2/iso (TR 1900 ms, TE 3.37 ms, TI 1100 ms, flip angle 15, slice thickness 1 mm).

#### 2.4.2. Analysis

All available MRI sequences were visually evaluated to exclude pre-existing non-neurodegenerative pathology (both focal and diffuse).

Then, T1W isotropic images were morphometrically analyzed using the FreeSurfer v6.0 software package (Harvard, MA, USA, http://surfer.nmr.mgh.harvard.edu/, accessed on 1 October 2018) on a single computer running the Linux CentOS 7 operating system. Detailed descriptions of core processes involved in FreeSurfer morphometric analysis are provided in previous publications [35,36,37,38,39,40,41,42].

Analysis was performed by using a recon-all script on the T1W MPRAGE 1 mm slice thickness images to produce global and regional cortical thickness and deep gray matter structure volume measurements. We chose to evaluate regional cortical thickness rather than regional cortical volume as it was shown to be more robust and less dependent on head size [43]. Cortical parcellation was performed according to the Desikan–Killiany atlas [44]. Region of interest selection was based on the previously mentioned models of the human olfactory system and included cortical and deep gray matter regions containing areas that were either described as structurally or functionally involved in olfactory processing [12,13,14,15,16] or shown to be associated with olfactory function in previously mentioned studies of the three populations [17,18,19,20,21,22,23,24,25,26,27,28,29], and available for segmentation in FreeSurfer software. Measures extracted for further analysis were as follows: volumes of the thalamus proper, caudate nucleus, putamen, pallidum, amygdala, and accumbens area; thicknesses of caudal and rostral anterior cingulate, posterior cingulate, cingulate isthmus, entorhinal, fusiform, parahippocampal, temporal pole, transverse temporal, inferior temporal, middle temporal, superior temporal, banks of the superior temporal sulcus, medial and lateral orbitofrontal, frontal pole, opercular, triangular, and orbital parts of the inferior frontal gyrus, precentral, postcentral, superior frontal, rostral middle frontal, supramarginal, and insular cortices. Hippocampal subfields were not included due to recently raised concerns about the validity of their measurements when derived from images acquired with 1 mm slice thickness [45]. In total there were 62 regions of interest (31 per hemisphere). Each subjects’ segmentation and parcellation output was visually inspected using the FreeView functionality to ensure no gross errors.

### 2.5. Statistical Analysis

Statistical analysis was performed with IBM SPSS version 22 (Armonk, NY, USA) and Microsoft Excel software packages.

Data for continuous variables were tested for normality using the Shapiro–Wilk test. Categorical variables were expressed as frequencies (%), while continuous variables were expressed as mean and standard deviation (SD).

ANOVA was used to compare the three groups’ continuous clinical and demographic variables (age, length of education, SS12 score, MMSE score) and global gray matter morphometric measures (cortical thickness and subcortical gray matter volume); in post-hoc analysis, pairwise comparisons were made with the Bonferroni correction. The Pearson Chi-squared test was used to compare the gender distribution. These general comparisons were made to assess the broad differences between our studied populations.

Correlation analyses were performed using the Pearson’s correlation coefficient (r) when both variables were normally distributed, and Spearman’s correlation coefficient (ρ) when only one or none of the variables were normally distributed. First, correlations between SS12 scores, MMSE scores, and age, and correlations between SS12 scores and global gray matter morphometric measures were assessed to detect possible associations between odor identification performance, cognitive performance, age, and global gray matter changes. Then, correlation analyses of SS12 scores and regional gray matter morphometric measures were carried out. For those, multiple comparison correction was implemented according to the false discovery rate (FDR) procedure, using the Benjamini–Hochberg two-stage sharpened method, with a maximum acceptable FDR set at 0.05, performed in a Microsoft Excel tool published in previous literature [46,47]. FDR-adjusted statistically significant regional correlations were further analyzed for the elimination of global confounding variables: SS12 scores were adjusted for age (because the majority of patients belonged to the same >60 year old group which would not allow to take into consideration differences in age within this group), and MMSE score (because general cognitive impairment may also diminish odor identification performance), while regional cortical thicknesses were adjusted for age, MMSE score, and global cortical thickness (to separate the effect of age, cognitive impairment, and generalized gray matter atrophy from the effect of regional gray matter atrophy). Finally, regional correlation analysis was repeated for the adjusted variables, again implementing multiple comparison correction according to the FDR procedure, this time using the Benjamini–Hochberg less conservative graphically sharpened method [47,48].

## 3. Results

### 3.1. Clinical and Demographic Characteristics

The clinical and demographic characteristics of patients with AD, PD, and control subjects are summarized in Table 1. Two AD and two PD patients had an SS12 score of zero, but they were not outliers. One participant was a relative outlier in the control group with a low SS12 score of three and was removed from further analysis. While the age of AD and PD groups did not differ significantly compared with control subjects, AD patients were significantly older than PD patients (72.3 and 64.1 years, respectively; *p* = 0.012). Compared with controls, both AD and PD patients had lower SS12 scores (means of 9.6, 4.5 and 6.2, respectively; age-adjusted *p* < 0.001, F_2,57_ = 19.58, *p* < 0.001). While AD patients had slightly lower SS12 scores than PD patients, the difference was not statistically significant. AD patients had lower MMSE scores compared with PD patients and controls (means of 17.60, 28.50 and 29.26, respectively; age adjusted *p* < 0.001, F_2,57_ = 77.17, *p* < 0.001).

**Figure 1:**
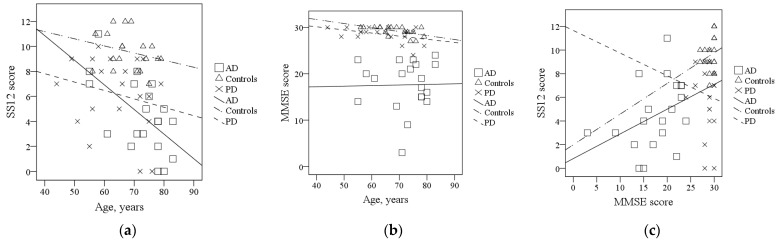
Correlations between SS12 scores, MMSE scores, and age: (**a**) Correlations between age and SS12 scores: AD: ρ = −0.526, *p* = 0.017; PD: ρ = −0.217, *p* = 0.359; Controls: r = –0.257, *p* = 0.288; (**b**) correlations between age and MMSE scores: AD: ρ = 0.086, *p* = 0.718; PD: ρ = −0.304, *p* = 0.193; Controls: ρ = −0.598, *p* = 0.007; (**c**) correlations between MMSE and SS12 scores: AD: ρ = 0.443, *p* = 0.050; PD: ρ = −0.114, *p* = 0.633; Controls: ρ = 0.203, *p* = 0.406. Abbreviations: AD, Alzheimer’s disease; PD, Parkinson’s disease; SS12, Sniffin’ Sticks 12 test; MMSE, Mini Mental State Examination.

Scatterplots of the correlations between SS12 scores, MMSE scores, and age are presented in Figure 1. The decline in SS12 scores was significantly age dependent only in the AD group (ρ = −0.526, *p* = 0.017). MMSE scores declined with age in the control group (ρ = −0.598, *p* = 0.007).

### 3.2. Global Gray Matter Morphometric Measures

Average global cortical thickness was lower in the AD group compared to PD (means of 2.16 and 2.38 mm, respectively; *p* = 0.005) and control (means of 2.16 and 2.38 mm, respectively; *p* < 0.001) groups (F_2,56_ = 13.34; *p* < 0.001). Average subcortical gray matter volume was lower in the AD group compared with PD (means of 49.61 and 55.05 cm^3^, respectively; *p* = 0.013; F_2,56_ = 4.57; *p* = 0.014). The differences in global gray matter morphometric measures between groups are shown in Table 2.

**Figure 2:**
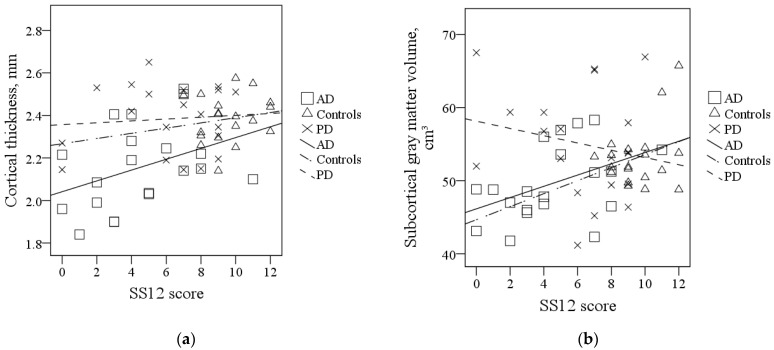
Correlations between SS12 scores and global gray matter morphometric measures: (**a**) Correlations between SS12 scores and cortical thickness: AD: r = 0.374, *p* = 0.104; PD: ρ = 0.008, *p* = 0.972; Controls: r = 0.166, *p* = 0.496; (**b**) Correlations between SS12 scores and subcortical gray matter volume: AD: r = 0.439, *p* = 0.053; PD: ρ = −0.140, *p* = 0.556; Controls: ρ = 0.062, *p* = 0.802. Abbreviations: AD, Alzheimer’s disease; PD, Parkinson’s disease; SS12, Sniffin’ Sticks 12 test.

Among AD patients, the correlation between SS12 scores and subcortical gray matter volume was of borderline significance (r = 0.439, *p* = 0.053). Among PD patients and the healthy elderly, SS12 scores and global gray matter morphometric measures were not correlated. Scatterplots of the correlations between SS12 scores and global gray matter morphometric measures are presented in Figure 2.

### 3.3. Regional Gray Matter Morphometric Measures

#### 3.3.1. AD Group

Among patients with AD there were 27 statistically significant (uncorrected) correlations between lower SS12 scores and regional gray matter atrophy; of those, 19 remained significant after multiple comparison correction with FDR and these are shown in Table 3.

Lower SS12 scores were moderately correlated with decreased cortical thickness in the following regions: bilateral medial and lateral orbitofrontal, bilateral orbital parts of the inferior frontal gyri, bilateral frontal and temporal poles, bilateral insula, bilateral rostral anterior cingulate, right isthmus cingulate, left entorhinal, and left superior frontal. Lower SS12 scores were also correlated with lower volumes of the right thalamus and left accumbens area.

**Figure 3:**
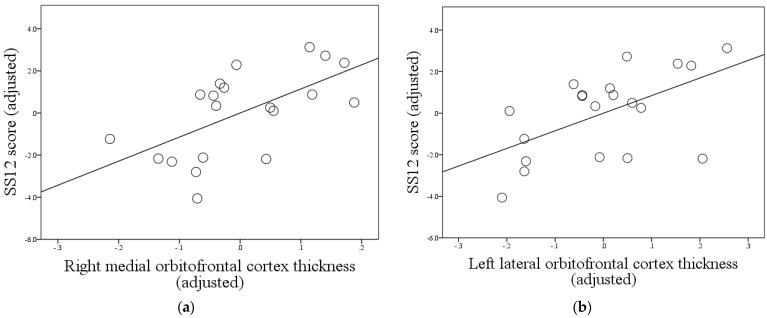
Correlations between SS12 scores and regional gray matter atrophy in patients with Alzheimer’s disease, after adjustment for age, MMSE score, and global cortical thickness: (**a**) correlation between SS12 score and right medial orbitofrontal cortex thickness: r = 0.601, *p* = 0.00504, p_adj_ = 0.049; (**b**) correlation between SS12 score and left lateral orbitofrontal cortex thickness: r = 0.562, *p* = 0.00984, p_adj_ = 0.049. SS12 scores are adjusted for age and MMSE score. Regional cortical thicknesses adjusted for age, MMSE score, and global cortical thickness. Multiple comparison correction performed according to the FDR procedure using the Benjamini–Hochberg graphically sharpened method, with a maximum acceptable FDR set at 0.05. Correlation graphs for regions with FDR-adjusted *p* > 0.05 not shown; 19 comparisons performed in total. Abbreviations: SS12, Sniffin’ Sticks 12 test; MMSE, Mini Mental State Examination; FDR, False Discovery Rate.

Following adjustment for possible confounding variables (age, MMSE score, and global cortical thickness), lower SS12 scores remained moderately correlated only with decreased thickness of the right medial orbitofrontal (r = 0.601, *p* = 0.00504, p_adj_ = 0.049) and left lateral orbitofrontal (r = 0.562, *p* = 0.00984, p_adj_ = 0.049) cortices (Figure 3).

#### 3.3.2. PD Group

Among patients with PD there were no statistically significant correlations between SS12 scores and regional gray matter morphometric measures.

#### 3.3.3. Control Group

Among the control group there were no statistically significant correlations between SS12 scores and regional gray matter morphometric measures.

## 4. Discussion

In this study we employed a unified design to perform three identical analyses among patients with AD and PD and the healthy elderly to identify possible associations between odor identification performance and regional deep and cortical gray matter morphometric measures. First, odor identification performance was significantly worse in the AD and PD groups than in the healthy elderly; also, AD patients performed slightly worse than PD patients, but the difference was not statistically significant. Second, in the AD group there were multiple positive correlations between odor identification performance and gray matter morphometric measures of regions known to be involved in olfactory processing, however, after correction for possible confounders, associations involving only two morphometric measures (thicknesses of right medial and left lateral orbitofrontal cortex thickness) remained significant. Finally, in the PD and healthy elderly groups, we demonstrated a lack of similar correlations between odor identification performance and regional gray matter morphometric measures.

First, the findings of decreased odor identification performance in patients with AD and PD compared with the healthy elderly confirm previous findings that this higher-order olfactory function is impaired in both neurodegenerative conditions. The slightly (but not statistically significantly) worse odor identification performance among patients with AD is also in line with previous findings that higher-order olfactory function impairment is present in both AD and PD but is relatively more pronounced in AD [1,6,7]. We also found age-related odor identification performance decline among patients with AD, which may indicate both the direct effect of aging, and the effect of more advanced AD pathology in older patients.

Second, findings in AD patients were mostly in line with previous reports about which regions are involved in olfactory information processing [12,13,14,15,20]. We showed significantly decreased odor identification performance and associated atrophy of the bilateral orbitofrontal cortices, which are consistently described as either primary or secondary olfactory areas. Other regional associations that were significant after correction for multiple comparisons but before correction for confounders might have suffered due to a small sample size, and may have remained significant in a larger cohort because those regions were also shown to be involved in olfactory processing in previously mentioned studies. Furthermore, our AD cohort was on average moderately cognitively impaired and had signs of global gray matter atrophy. Hence, associations between impaired odor identification and atrophy of numerous gray matter regions that were present before correction for global confounders may have represented the effect of broader gray matter atrophy involving the temporal, frontal, and limbic cortical areas. This broader atrophy may have contributed to global cognitive impairment that may have in turn negatively influenced higher-order olfactory processing. Thus, our findings may suggest that adequate odor identification performance could be impeded by disruption along the whole olfactory-cognitive pathway, either regionally in the olfactory system itself, or more globally in the widespread areas involved with various levels of cognitive processing (memory, emotion, sensorimotor aspects of speech, planning, executive function). Definitively differentiating which component (strictly regional olfactory or a broader general cognitive) plays the most significant role in a moderately cognitively impaired AD patient cohort is beyond the scope of this structural MRI study. However, correlations involving the bilateral orbitofrontal cortices were the strongest and remained significant when controlling for the previously mentioned confounders representing global cognitive impairment and structural gray matter changes. Therefore, our findings support the key role of the orbitofrontal cortex in odor identification performance among patients with AD.

In contrast, among PD patients there were no similar positive statistically significant associations between odor identification performance and regional cortical or deep gray matter morphometric measures, even though there are multiple previous reports of such positive associations [24,25,26,27]. Also, we did not find opposite (negative) associations between odor identification performance and regional gray matter morphometric measures that were reported in one study suggesting the cognitive reserve hypothesis as an explanation for their findings [29]. Taken together, our lack of significant associations between impaired odor identification performance and any regional gray matter changes among patients with PD seems to lie between these two patterns of relationships reported in the literature.

There are several possible explanations for different findings among AD and PD patients with almost similarly impaired odor identification performance.

First, the findings may represent a difference in the strength of relationships between odor identification and regional gray matter atrophy among these populations. Given that the sample sizes of AD and PD patients were identical, both groups were evaluated with identical methods, and we detected significant associations only in AD patients, it could be that similar associations (which were previously reported in the literature) existed in our PD patients but were weaker and beyond the statistical and methodological power of our study. For example, one study which reported decreased orbitofrontal cortex volume to be associated with impaired olfactory performance in patients with PD had a sample size of 40, twice our 20 [24]. Thus, our study offers additional information by investigating identically sized samples of these populations with identical methods and suggesting that there may be differences among them in the strength of associations between odor identification performance and regional gray matter atrophy, namely, that these associations may be more pronounced in patients with AD.

Other possible explanations for our findings are varied and may involve relationships of a different nature or location. One study showed that while in AD diminished olfactory performance was associated with cortical atrophy, in Lewy body-related cognitive decline it was white matter abnormalities that played a significant role in olfactory dysfunction [21]; this may explain the lack of structural gray matter findings among our patients with PD. Also, olfactory impairment could have been caused by damage and decreased volumes of the olfactory bulbs or piriform cortices, which are reported to be involved in PD-related olfactory loss but were not segmented in our analysis [28,49,50]. Furthermore, while we did not detect significant gray matter atrophy using structural MRI, PD patients with worse olfactory performance could have disrupted functional connectivity between key olfactory regions that would reduce their odor identification performance and could be detectable by functional MRI [51].

Finally, the lack of significant associations between odor identification performance and regional gray matter atrophy among the healthy elderly may be due to a combination of lower sample and effect sizes. Indeed, regional atrophy of the orbitofrontal cortex was reported to be associated with impaired olfactory performance in the healthy elderly in a study with a significantly larger sample size [52]. Our findings may be interpreted to show that this association among the healthy elderly, if present, is significantly weaker when compared with AD patients, as it was only detected in the latter group.

Our study had limitations. PD patients were selected based on the United Kingdom Parkinson’s Disease Society Brain Bank Clinical Diagnostic Criteria, and not the more recent Movement Disorder Society Clinical Diagnostic Criteria. As was discussed previously, the sample sizes of each group were small, which would allow to detect only relatively large effect sizes. Also, several characteristics of the AD group could have introduced confounding effects: first, AD patients on average were moderately cognitively impaired, which made differentiating between true odor identification dysfunction and a global cognitive impairment difficult; second, AD patients had decreased global gray matter morphometric measures when compared with patients with PD and the healthy elderly; finally, while the healthy elderly control group was chosen to be age- and sex-matched to both the AD and PD patients, they, themselves, differed significantly in that AD patients were older than PD patients. We corrected for these possible confounders (MMSE score, global cortical thickness, and age) in our regional correlation analysis of AD patients. The possible effect of the relatively younger age of the PD group on the lack of significant correlations among them should be lessened by the fact that our PD patients were older than PD patients in whom significant associations between olfactory performance and structural gray matter changes were previously detected in the literature [24]. Finally, the unavailability of the complete primary olfactory system segmentation (anterior olfactory nucleus, olfactory tubercle, and piriform cortex) resulted in our study being more focused on the secondary and higher-order olfactory regions, and areas with which they were reported to be connected, while only parts of the primary olfactory system were included in our analysis.

Further studies employing a similar unified study design with larger cohorts, more comprehensive olfactory testing, combined structural and functional imaging protocols, and a complete human olfactory system segmentation would be needed to better understand and compare how distinct neurodegenerative pathologies and healthy aging affect various facets of olfactory performance, especially explore the possible differences in the relationships between olfactory performance and its regional structural and functional correlates, and the implications of such differences for understanding olfactory neurodegenerative processes.

## 5. Conclusions

In conclusion, we have shown that while odor identification performance was impaired in both AD and PD patients, correlations between this impairment and regional gray matter atrophy were detected only in AD patients, specifically involving the thicknesses of bilateral orbitofrontal cortices. These findings support the key role of the orbitofrontal cortex in odor identification among patients with AD, and suggest that associations between impaired odor identification performance and regional gray matter atrophy may be relatively more pronounced in AD rather than in PD.

## Figures and Tables

**Table 1:** Clinical and demographic characteristics.

	AD (*n* = 20)	PD (*n* = 20)	Controls (*n* = 19)	ANOVA F_2,57_; *p*	Group Differences
Age in years, mean (SD), range	72.30 (8.74), 28	64.05 (10.13), 35	68.11 (6.78), 23	F = 4.5; *p* = 0.015	AD > PD, *p* = 0.012 ^a^
Gender, (Male:Female)	11:9	11:9	11:8	χ^2^ = 2.3 *p* = 0.893 ^b^	
Education in years, mean (SD)	12.95 (3.65)	14.65 (2.94)	14.95 (3.19)	F = 2.14; *p* = 0.127	
SS12 score, mean (SD)	4.50 (2.91)	6.20(2.98)	9.60 (1.50)	F = 19.58; *p* < 0.001	AD < C, *p* < 0.001 ^a^ PD < C, *p* < 0.001 ^a^
MMSE score, mean (SD)	17.60 (5.35)	28.50(1.57)	29.26 (1.05)	F = 77.17; *p* < 0.001	AD < C, *p* < 0.001 ^a^ AD < PD, *p* < 0.001 ^a^

^a^ Pairwise comparison with Bonferroni correction; ^b^ χ^2^ test. Abbreviations: AD, Alzheimer’s disease; PD, Parkinson’s disease; C, controls; SS12, Sniffin‘ Sticks 12 test; MMSE, Mini Mental State Examination; SD, standard deviation.

**Table 2:** Differences in global gray matter morphometric measures.

.	Group	Mean (SD)	ANOVA F_2,56_; *p*	Group Differences
Cortical thickness, mm	AD	2.16 (0.20)	F_2,56_ = 13.34; *p* < 0.001	AD < PD; *p* < 0.001 ^a^
	PD	2.38 (0.16)		AD < C; *p* < 0.001 ^a^
	C	2.38 (0.11)		
Subcortical gray matter volume, cm^3^	AD	49.61 (5.11)	F_2,56_ = 4.57; *p* = 0.014	AD < PD; *p* = 0.013 ^a^
	PD	55.05 (7.42)		
	C	53.18 (4.27)		

^a^ Pairwise comparison with Bonferroni correction. Abbreviations: AD, Alzheimer’s disease; PD, Parkinson’s disease; C, controls; SD, standard deviation.

**Table 3:** Statistically significant correlations between lower SS12 scores and regional gray matter atrophy in patients with Alzheimer’s disease, without adjustment for possible confounders.

Gray Matter Region	Correlation with SS12 Score	Uncorrected *p* Value	FDR-Adjusted *p* Value ^a^
Right medial orbitofrontal cortex thickness	0.673	0.00115	0.034
Left lateral orbitofrontal cortex thickness	0.649	0.00196	0.034
Right pars orbitalis cortex thickness	0.623	0.00336	0.034
Right frontal pole cortex thickness	0.620	0.00353	0.034
Right thalamus proper volume	0.604	0.00480	0.034
Right lateral orbitofrontal cortex thickness	0.599	0.00525	0.034
Left medial orbitofrontal cortex thickness	0.595	0.00561	0.034
Left temporal pole cortex thickness	0.594	0.00575	0.034
Right insular cortex thickness	0.592	0.00592	0.034
Left accumbens area volume	0.503	0.00611	0.034
Right temporal pole cortex thickness	0.587	0.00675	0.034
Left rostral anterior cingulate cortex thickness	0.579	0.00750	0.034
Left pars orbitalis cortex thickness	0.573	0.00827	0.036
Left insular cortex thickness	0.569	0.00877	0.036
Right isthmus cingulate cortex thickness	0.553	0.01100	0.043
Left entorhinal cortex thickness	0.537	0.01470	0.048
Right rostral anterior cingulate cortex thickness	0.536	0.01481	0.048
Left frontal pole cortex thickness	0.533	0.01554	0.048
Left superior frontal cortex thickness	0.531	0.01592	0.048

^a^ Adjusted for multiple comparisons according to the FDR procedure using the Benjamini–Hochberg two-stage sharpened method, with a maximum acceptable FDR set at 0.05. Regions with FDR-adjusted *p* > 0.05 not shown; 62 comparisons performed in total. Among the shown correlations, all except right isthmus cingulate cortex thickness were evaluated with the Pearson’s correlation coefficient. Abbreviations: SS12, Sniffin’ Sticks 12; FDR, False Discovery Rate.

## Data Availability

Data presented in this study are available on request from the corresponding author.
